# Effectiveness of dapagliflozin on vascular endothelial function and glycemic control in patients with early-stage type 2 diabetes mellitus: DEFENCE study

**DOI:** 10.1186/s12933-017-0564-0

**Published:** 2017-07-06

**Authors:** Fumika Shigiyama, Naoki Kumashiro, Masahiko Miyagi, Kayoko Ikehara, Eiichiro Kanda, Hiroshi Uchino, Takahisa Hirose

**Affiliations:** 1 Division of Diabetes, Metabolism, and Endocrinology, Department of Medicine, Toho University Graduate School of Medicine, 6-11-1 Omori-Nishi, Ota-ku, Tokyo, 143-8541 Japan; 2 Department of Nephrology, Tokyo Kyosai Hospital, Tokyo, Japan; 3 Life Science and Bioethics Research Center, Tokyo Medical and Dental University, Tokyo, Japan

**Keywords:** Dapagliflozin, Endothelial function, Type 2 diabetes

## Abstract

**Background:**

Recent studies reported that sodium glucose cotransporter 2 (SGLT2) inhibitors can potentially reduce the risk of cardiovascular mortality in patients with type 2 diabetes mellitus (T2DM). However, there is little or no information on the therapeutic effects of SGLT2 inhibitors on the progression of atherosclerosis. This dapagliflozin effectiveness on vascular endothelial function and glycemic control (DEFENCE) study was designed to determine the effects of dapagliflozin, a SGLT2 inhibitor, on endothelial function in patients with early-stage T2DM.

**Methods:**

DEFENCE is a prospective, randomized, open-label, blinded-endpoint, parallel-group, comparative clinical trial. Between October 2015 and August 2016, 80 T2DM patients treated with 750 mg of metformin (hemoglobin A1c ≥6.0 and <8.0%, n = 80) were enrolled and randomized to receive either 1500 mg/day metformin (the metformin group, n = 40), or 750 mg/day metformin supplemented with 5 mg/day dapagliflozin (the dapagliflozin group, n = 40), for 16 weeks. The primary endpoint was a change in flow-mediated dilation (FMD) from baseline to the end of the 16-week treatment period. The secondary outcomes include changes in indexes of glycemic control, lipid metabolism, and oxidative stress, body composition, and safety evaluation.

**Results:**

Although FMD tended to improve only in the dapagliflozin group, ΔFMD was comparable between the two groups. Analysis of patients with HbA1c >7.0% showed significant improvement of FMD in the dapagliflozin group than metformin group (P < 0.05). HbA1c, fasting plasma glucose, plasma glucagon, and body weight significantly decreased in both groups. Interestingly, urine 8-hydroxy-2′-deoxyguanosin, a biomarker of oxidative stress, was significantly lower in the dapagliflozin group than metformin group at 16 weeks (P < 0.001).

**Conclusions:**

Dapagliflozin add-on therapy to metformin for 16 weeks improved endothelial function, as assessed by FMD, in patients with inadequately controlled early-stage T2DM. Improvement in oxidative stress may contribute to the improvement in FMD.

*Trial registration* University Hospital Medical Information Network Clinical Trial Registry (UMIN000018754)

**Electronic supplementary material:**

The online version of this article (doi:10.1186/s12933-017-0564-0) contains supplementary material, which is available to authorized users.

## Background

Type 2 diabetes mellitus (T2DM) is a major risk factor for the progression of atherosclerosis and development of cardiovascular diseases [1, 2]. It is reported that the prevalence of coronary heart disease is two to fourfolds higher in T2DM patients compared to non-diabetes patients [3]. The risk of cardiovascular diseases progressively increases at the stage of impaired glucose tolerance and/or post-prandial hyperglycemia [4, 5]. The UKPDS study concluded that treatment of T2DM at an early stage significantly reduces the incidence of macroangiopathy [6]. This is conceivable since it is difficult to improve endothelial function after the progression of atherosclerosis. Thus, early screening for atherosclerosis is important in the prevention of future cardiovascular events. The initial stages of atherosclerosis can be detected at present by using various non-invasive devices. Among them, the flow-mediated dilation (FMD) method can measure endothelial function and predict the prognosis of cardiovascular events [7].

Various oral glucose-lowering agents with different mechanisms of action are currently available in the market. The American Diabetes Association and the European Association for the Study of Diabetes recommend the use of metformin as the first-line drug [8–10]. Metformin has been reported to prevent cardiovascular events in obese patients [8]. Inhibitors of sodium glucose cotransporter 2 (SGLT2), a new family of oral glucose-lowering agents, prevent a rise in blood glucose level by suppressing renal reabsorption of sodium and glucose, and by enhancing urinary glucose excretion [11]. Dapagliflozin was the first SGLT2 inhibitor introduced to the world market. SGLT2 inhibitors have additional effects beyond lowering blood glucose, such as helping reduce body weight [12], lower blood pressure [13], and reduce serum triglyceride level [14]. It has also been reported that SGLT2 inhibitors, through their blood pressure lowering effects, can improve arterial stiffness [15]. Thus, SGLT2 inhibitors seem to have multiple metabolic and cardiovascular benefits. However, only a few large cardiovascular outcome studies designed to elucidate the effect of SGLT2 inhibitors have been conducted [16, 17]. These include a currently ongoing clinical trial on dapagliflozin [Dapagliflozin Effect on CardiovascuLAR Events (DECLARE TIMI-58); NCT01730534]. Recently, the EMPA-REG outcomes trial investigated the effects of empagliflozin, another SGLT2 inhibitor [17], and reported that empagliflozin significantly reduced the all-cause and cardiovascular mortality rates and the rate of hospitalization for heart failure compared to placebo in T2DM patients with high cardiovascular risk [17]. Similarly, dapagliflozin may lower blood glucose levels and at the same time prevent cardiovascular events. The clinical trial regarding a possible favorable action of dapagliflozin on heart failure, the REFORM study [18], is ongoing and we should expect for the results. However, there is no information on whether SGLT2 inhibitors can halt the progression of early atherosclerosis as primary prevention.

The present clinical trial [dapagliflozin effectiveness on vascular endothelial function and glycemic control in T2DM (DEFENCE)] is the first study designed to assess the anti-atherosclerotic effects of dapagliflozin in early-stage T2DM patients, using FMD as a surrogate marker for cardiovascular events. The study also compared the effects of dapagliflozin add-on to those of metformin alone on various metabolic markers.

## Methods

### Study design

The DEFENCE study is a prospective, randomized open-label, blinded-endpoint, parallel-group, comparative study, registered with the University Hospital Medical Information Network Clinical Trial Registry (UMIN000018754), a non-profit organization in Japan that meets the requirements of the International Committee of Medical Journal Editors (ICMJE). The study was approved by the Medical Ethics Committee of Toho University (approval #27249) and conducted according to the Declaration of Helsinki and current legal regulations in Japan. The processes of enrollment, randomization, data collection and management were conducted by third-party entities to secure non-bias.

### Study population

A total of 80 Japanese patients with T2DM who regularly visited the Outpatient Clinics of 15 institutions in Japan (listed under Additional file 1) participated in the study. The inclusion criteria were as follows: (1) type 2 diabetes patients who have been treated for more than 12 weeks with 750 mg of metformin or one type of oral glucose-lowering agent in addition to 750 mg of metformin (in the case of sulfonylurea users, <2 mg of glimepiride or <40 mg of gliclazide were allowed), in addition to diet and exercise; (2) hemoglobin A1c (HbA1c) (National Glycohemoglobin Standardization Program; NGSP) level of ≥6.0 to <8.0%; (3) males and females aged 20–74; (4) patients who could be monitored closely for medication compliance; (5) signing written consent form to participate in the study. The following criteria were used to exclude subjects from the study: (1) patients with type 1 diabetes or secondary diabetes; (2) patients who, within 12 weeks before signing the consent form, had used SGLT2 inhibitors, glucagon-like peptide-1 agonists, or insulin; (3) patients who, within 12 weeks before signing the consent, had used a dose of metformin exceeding 750 mg/day; (4) patients who, within 12 weeks before consent, had started taking angiotensin-converting enzyme inhibitor (ACE inhibitor), angiotensin II receptor antagonist (ARB), HMG-CoA reductase inhibitor (statin), or antiplatelet drugs, or had the dose changed (including reduction of the dose); (5) patients with severe infection, were scheduled for surgery, or suffered serious trauma recently; (6) patients with history of myocardial infarction, angina, stroke or cerebral infarction; (7) patients with atrial (chronic) fibrillation, frequent supraventricular or ventricular ectopy; (8) patients with moderate or severe cardiac insufficiency (those with class III or more as classified by the NYHA/New York Heart Association); (9) patients with ankle brachial pressure index of <0.9; (10) patients with serious liver or renal functional failure [serum creatinine ≥1.3 mg/dL or estimated glomerular filtration rate (eGFR) of <45 mL/min/1.73 m^2^]; (11) patients who, within 12 weeks before consent, had unstable blood pressure, or lipid abnormalities; (12) patients dependent on alcohol or illicit drugs; (13) female patients who were pregnant or breastfeeding, possibly pregnant, or planning to become pregnant within the study period; (14) patients with dehydration (abnormal test results of hematocrit and BUN values, and complaint of symptoms of dehydration); (15) patients on diuretics; (16) patients with urinary tract or genital infections within 12 weeks before consent; (17) patients with history of hypersensitivity to the study drugs; (18) patients with severe ketosis, diabetic coma or precoma; (19) patients considered unsuitable subjects by the attending physician; (20) patients with an implanted pacemaker.

### Randomization and study intervention

The eligible subjects were randomly assigned in equal numbers into two groups; the metformin group: the dose of metformin was increased from 750 to 1500 mg/day, and the dapagliflozin group: 5 mg/day dapagliflozin was added to 750 mg/day metformin. The randomization was achieved by a computer-based dynamic allocation method based on the with/without administration of statins and dipeptidyl peptidase 4 (DPP-4) inhibitors. After enrollment in the study, all patients were prohibited from changing the dose of concomitant drugs or adding any other drugs, such as other glucose-lowering agents, anti-hypertension drugs, lipid-lowering and antiplatelet agents. Baseline measurements of blood and urine variables and FMD were performed during the 4–6 weeks of the screening period. After baseline data collection, the assigned therapies were started. The treatment intervention date was set as the study start date (metformin group: the day of increasing the dose of metformin; dapagliflozin group: the day of addition of dapagliflozin to metformin). The assigned treatment was continued for 16 weeks (duration of the study).

### Recorded variables and schedule

Clinical and biochemical data were collected at baseline and after the 16-week treatment period. The FMD was conducted at Toho University Omori Medical Center. All blood tests were carried out after overnight fast. Measurement of the following parameters was outsourced to the central laboratory (SRL Laboratory, Tokyo, Japan): HbA1c, fasting plasma glucose (FPG), C-peptide, plasma insulin, glucagon, total cholesterol, high-density lipoprotein (HDL) cholesterol, low-density lipoprotein (LDL) cholesterol, triglyceride, adiponectin, apolipoprotein B48 and urinary 8-hydroxy-2′-deoxyguanosin (8-OHdG). The homeostatic model assessment of insulin resistance (HOMA-IR) was calculated from the obtained data. In addition to these parameters, other biochemical safety parameters (e.g. red blood cell count, hematocrit, and uric acid) were measured.

### Flow-mediated dilation

The FMD was measured using the UNEX EF38G (UNEX Corporation, Nagoya, Japan) by a technologist who was not a participant in the study and was blinded to the study groups. The protocol and methodology have been described in detail previously [19–21]. Briefly, all measurements were performed under fasting and non-smoking conditions in the early morning in a temperature-controlled room (25 °C). After resting for at least 15 min, the pressure cuff was placed on the forearm to capture baseline images of the brachial artery using high-resolution ultrasound. Then, the cuff was inflated and kept at 50 mmHg above the systolic blood pressure to occlude the brachial artery. The cuff was released 5 min later, and the image of the brachial artery was captured. The diameters of the brachial artery on the pre- and post-hyperemia images were used to calculate changes in FMD according to the following formula: [FMD (%) = (maximum diameter − diameter at rest) × 100/diameter at rest].

### Study outcome

The primary study outcome was a change in FMD [ΔFMD (=value at week 16 − value at baseline)]. The secondary endpoints included changes in the values of the following items at the end of the 16-week treatment, relative to the baseline: (1) indexes of glycemic control: HbA1c, FPG level, C-peptide, plasma insulin, glucagon and HOMA-IR; (2) indexes of lipid metabolism: total cholesterol, HDL cholesterol, LDL cholesterol and triglyceride; (3) indexes of atherosclerosis and oxidative stress: adiponectin, apolipoprotein B48, and urinary 8-OHdG.

### Safety and evaluation of adverse events

During the course of the study, the investigators constantly monitored the appearance of any hypoglycemic or hyperglycemic symptoms and signs and all other adverse events (AEs) through regular medical checkups. When AEs occurred, details were reported immediately to the respective institution, the principal investigator and the administration office. All related AEs, not only side effects to the drug, but also abnormal values from the clinical tests, were reported and documented.

### Sample size and statistical analysis

Due to the lack of previous reports on ΔFMD with SGLT2 inhibitor therapy, ΔFMD under treatment with DPP-4 inhibitor were used as reference for sample size calculation. It has been reported that 12-week treatment with sitagliptin (DPP-4 inhibitor) produced a mean ∆FMD of 1.69 ± 1.76% (±SD) [22]. Accordingly, we assumed that a similar ∆FMD would be expected after 16-week dapagliflozin therapy. Furthermore, another study reported that 24-week metformin therapy produced ∆FMD of 0.72 ± 1.52% [23]. Accordingly, we assumed that 16-week metformin treatment in this study would produce ∆FMD of 0.5 ± 1.52%. Thus, we assumed that the difference in ∆FMD after 16-week treatment between the two groups would be 1.19%. Based on these assumptions, the number of cases required to detect a significant difference in ∆FMD between the two groups under the conditions of two-sided *P* value of 5% and power of 85%, was 36 patients per group; with a total sample size of 72. Assuming a dropout rate of 10%, the target number of patients was therefore set to 40 cases per group, with a total of 80 cases.

Analyses of the primary and secondary endpoints were performed on the full analysis set (FAS). FAS includes subjects who were enrolled in this study and assigned to a study treatment, however, research subjects with a significant study protocol violation were excluded. Safety analysis with AE was performed on the treated set. Every subgroup analysis plan was pre-specified before the statistical analysis plan was determined.

The reported values were expressed as mean ± SD unless otherwise mentioned in the text. Statistical analyses were conducted with two-sided *P* value set at 5%. Summary statistics were executed for background data. The Fisher’s exact test was applied for nominal variables, and the Student’s *t* test was applied for continuous variables for comparisons between groups. The analysis plan resembled that applied in a previous study published by our group [21]. Briefly, for the primary endpoint, i.e., ∆FMD after 16-week treatment, we compared the fixed effects of the groups using analysis of covariance and covariates of the allocation adjustment factors (statin use and DPP-4i use). Analyses of the secondary endpoints were performed using Student’s *t* test for comparisons of two groups. Regarding safety information, a list of all AEs was prepared for each group and compared using Fisher’s exact test. All statistical analyses were performed by the administrative office of the DEFENCE study, with supervision by an independent statistician, using SAS software version 9.3 (SAS Institute Inc., Cary, NC).

### Human rights and ethical principles of study subjects

The study protocol complied with the “World Medical Association Declaration of Helsinki” (2013 revision), and “Ethical Guidelines for Medical and Health Research Involving Human Subjects” (December 22, 2014, Ministry of Education, Culture, Sports, Science and Technology/Ministry of Health, Labor and Welfare), and all other relevant laws and regulations.

## Results

### Clinical characteristics of the two groups

**Fig. 1:**
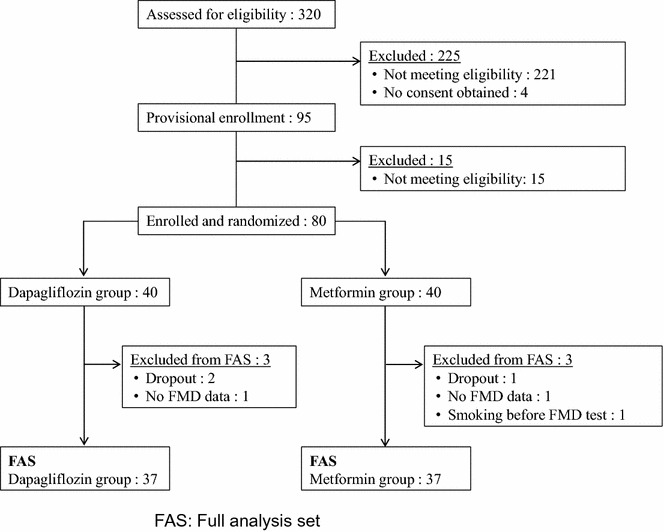
Recruitment process of patients. *FAS* full analysis set

A total of 80 patients were enrolled in this study between October 2015 and August 2016. They were randomized into the metformin group and the dapagliflozin group. Of the total, 74 patients completed the study and the FAS population included each 37 patients in the metformin group and dapagliflozin group, respectively (Fig. 1). Table 1 shows the baseline clinical characteristics of the patients. There were no significant differences in all the clinical characteristics between both groups. Notably, the mean duration of T2DM was around 6 years and the mean HbA1c was lower than 7% in both groups. Table 1 shows that only a small number of the study patients had diabetic or macrovascular complications.

**Table 1:** Patients’ characteristics

Characteristics	Dapagliflozin group	Metformin group	*P* value
Sex (male/female)	25 (67.6)/12 (32.4)	22 (59.5)/15 (40.5)	0.63
Age (years)	57.9 ± 8.3 (37)	59.4 ± 10.1 (37)	0.51
Duration of diabetes (years)	5.4 ± 4.4 (37)	6.3 ± 4.2 (37)	0.40
BMI (kg/m^2^)	26.8 ± 4.6 (37)	26.3 ± 3.5 (37)	0.60
Current smoking	10 (27.0)	6 (16.2)	0.50
Current alcohol	14 (37.8)	12 (32.4)	0.81
Diabetic retinopathy	6 (16.2)	2 (5.4)	0.26
Simple retinopathy	4 (10.8)	0 (0.0)	0.09
Preproliferative retinopathy	1 (2.7)	0 (0.0)	
Proliferative retinopathy	1 (2.7)	2 (5.4)	
Diabetic nephropathy	10 (27.0)	13 (35.1)	0.62
UACR 30–299 mg/g creatinine	9 (24.3)	13 (35.1)	0.45
UACR ≥300 mg/g creatinine	1 (2.7)	0 (0.0)	
Diabetic neuropathy	6 (16.2)	6 (16.2)	1.00
Polyneuropathy	3 (8.1)	0 (0.0)	0.13
Mononeuropathy	1 (2.7)	5 (13.5)	
Unknown	2 (5.4)	1 (2.7)	
Macrovascular complications	1 (2.7)	1 (2.7)	1.00
Cerebrovascular disease	0 (0.0)	0 (0.0)	–
Coronary disease	0 (0.0)	0 (0.0)	–
Peripheral arterial disease	1 (2.7)	1 (2.7)	1.00
Other complications	34 (91.9)	32 (86.5)	0.71
Renal disease	1 (2.7)	0 (0.0)	1.00
Liver disease	4 (10.8)	3 (8.1)	1.00
Hypertension	15 (40.5)	14(37.8)	1.00
Hyperlipidemia	25 (67.6)	23 (62.2)	0.81
HbA1c (NGSP%)	6.8 ± 0.5 (37)	6.9 ± 0.5 (37)	0.39
HbA1c (mmol/mol)	50.3 ± 5.5 (37)	51.4 ± 5.7 (37)	0.39
Fasting plasma glucose (mg/dL)	133.5 ± 27.1 (37)	139.6 ± 20.2 (37)	0.28
C-peptide (ng/mL)	2.1 ± 0.8 (37)	2.3 ± 1.1 (37)	0.34
Insulin (μIU/mL)	8.9 ± 5.5 (37)	10.3 ± 7.5 (37)	0.38
Glucagon (pg/mL)	164.6 ± 33.5 (37)	171.6 ± 42.4 (37)	0.43
Systolic blood pressure (mmHg)	129.2 ± 13.7 (37)	130.0 ± 12.4 (37)	0.78
Diastolic blood pressure (mmHg)	81.8 ± 9.6 (37)	79.9 ± 8.5 (37)	0.36
Total cholesterol (mg/dL)	201.8 ± 33.1 (35)	194.1 ± 31.9 (35)	0.33
HDL cholesterol (mg/dL)	50.9 ± 9.0 (35)	53.4 ± 15.4 (35)	0.42
LDL cholesterol (mg/dL)	109.6 ± 33.2 (35)	96.1 ± 25.0 (35)	0.06
Triglyceride (mg/dL)	142.8 ± 53.1 (37)	145.2 ± 69.0 (37)	0.87
Serum creatinine (mg/dL)	0.7 ± 0.1 (37)	0.7 ± 0.2 (37)	0.72
Uric acid (mg/dL)	5.6 ± 1.1 (37)	5.8 ± 1.2 (37)	0.41
Anti-diabetic drugs	37 (100.0)	37 (100.0)	–
Biguanides	37 (100.0)	37 (100.0)	–
DPP-4 inhibitors	6 (16.2)	7 (18.9)	1.00
Sulfonylureas	2 (5.4)	4 (10.8)	0.67
α-Glucosidase inhibitors	2 (5.4)	1 (2.7)	1.00
Glinides	2 (5.4)	4 (10.8)	0.67
Thiazolidinediones	0 (0.0)	0 (0.0)	–
Antihypertensive drugs	11 (29.7)	14 (37.8)	0.62
Diuretic drugs	0 (0.0)	1 (2.7)	1.00
Calcium channel blockers	6 (16.2)	10 (27.0)	0.40
ACE inhibitors	4 (10.8)	0 (0.0)	0.11
Angiotensin II receptor blockers	6 (16.2)	11 (29.7)	0.27
Direct renin inhibitors	0 (0.0)	0 (0.0)	–
β-Blockers	1 (2.7)	0 (0.0)	1.00
α-Blockers	1 (2.7)	0 (0.0)	1.00
Lipid-lowering agents	23 (62.2)	21 (56.8)	0.81
Statins	14 (37.8)	15 (40.5)	1.00
Fibrates	7 (18.9)	5 (13.5)	0.75
Ezetimibe	2 (5.4)	1 (2.7)	1.00
Probucol	1 (2.7)	0 (0.0)	1.00
EPAs	3 (8.1)	3 (8.1)	1.00
Resins	0 (0.0)	0 (0.0)	–
Antithrombotic agents	1 (2.7)	1 (2.7)	1.00
Antiplatelet agents	1 (2.7)	1 (2.7)	1.00
Anticoagulants	0 (0.0)	0 (0.0)	–

Data are number (%), mean ± standard deviation (*n*), or median [first quartile, third quartile] (*n*). *P* values by the *t* test or Wilcoxon rank sum test for continuous data, and by Fisher exact test for categorical data

*BMI* body mass index, *UACR* urinary albumin-to-creatinine ratio, *HbA1c* hemoglobin A1c, *NGSP* national glycohemoglobin standardization program, *HDL* high-*density* lipoprotein, *LDL* low-*density* lipoprotein, *DPP-4* dipeptidyl peptidase-4, *ACE* angiotensin-converting enzyme, *EPA* eicosapentaenoic acid

### Endothelial function after 16 weeks of treatment

**Fig. 2:**
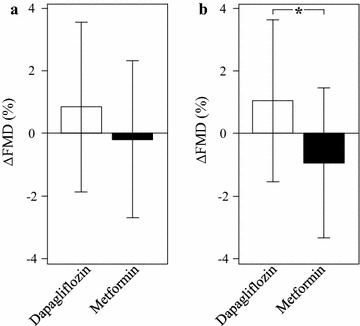
Effects of each treatment on ΔFMD. **a** ΔFMD in FAS. **b** ΔFMD in subpopulation of HbA1c at baseline ≥7.0%. *P < 0.05

The primary endpoint of this study was the ΔFMD after 16 weeks of treatment. Table 2 shows the FMD values at baseline, week 16 and the ΔFMD values. Although FMD tended to improve in the dapagliflozin group (P = 0.06), ΔFMD was comparable between the two groups in the FAS population (Table 2; Fig. 2a). Interestingly, however, subgroup analyses of patients with HbA1c ≥7.0% for ΔFMD showed that FMD improved significantly in the dapagliflozin group compared with the metformin group (P < 0.05, Table 2; Fig. 2b). The result of covariance analysis for ΔFMD after adding the baseline FMD also showed the significant difference between the two groups (P < 0.05).

**Table 2:** ΔFMD

	Dapagliflozin group	Metformin group	*P* value
FMD (%)			
FAS population			
Baseline	4.80 ± 1.86 (37)	5.37 ± 2.95 (37)	0.33
Week 16	5.66 ± 2.12 (37)	5.18 ± 2.09 (37)	0.33
Change	0.85 ± 2.71 (37)	−0.19 ± 2.51 (37)	0.09
*P* value within group	0.06	0.65	
Subpopulation: HbA1c at baseline <7.0%			
Baseline	4.72 ± 1.88 (24)	4.88 ± 2.76 (20)	0.83
Week 16	5.47 ± 2.42 (24)	5.33 ± 2.23 (20)	0.85
Change	0.75 ± 2.82 (24)	0.45 ± 2.48 (20)	0.71
*P* value within group	0.21	0.43	
Subpopulation: HbA1c at baseline ≥7.0%			
Baseline	4.95 ± 1.91 (13)	5.94 ± 3.14 (17)	0.30
Week 16	6.01 ± 1.43 (13)	5.01 ± 1.96 (17)	0.12
Change	1.05 ± 2.59 (13)	−0.94 ± 2.39 (17)	0.041
*P* value within group	0.17	0.13	

Data are mean ± standard deviation (*n*). *P* values show results of comparisons between groups by *t* test. *P* values within groups are results of paired *t* test

*FAS* full analysis set. See Table 1 for other abbreviations

### Comparable changes in glycemic control between the two groups

HbA1c and fasting plasma glucose improved significantly from baseline to 16-weeks in both groups (Table 3). Fasting plasma insulin decreased significantly only in the dapagliflozin group, while plasma glucagon level decreased in both groups. HOMA-IR, which is recognized as an index of insulin resistance, decreased significantly in both groups.

**Table 3:** Changes in parameters of glycemic control

Parameters	Dapagliflozin group	Metformin group	*P* value
HbA1c (%)			
Baseline	6.8 ± 0.5 (37)	6.9 ± 0.5 (37)	0.39
Week 16	6.5 ± 0.5 (37)	6.5 ± 0.6 (37)	0.76
Change	−0.2 ± 0.4 (37)	−0.4 ± 0.3 (37)	0.09
*P* value within group	0.001	<0.001	
HbA1c (mmol/mol)			
Baseline	50.3 ± 5.5 (37)	51.4 ± 5.7 (37)	0.39
Week 16	48.0 ± 5.4 (37)	47.5 ± 6.2 (37)	0.76
Change	−2.4 ± 4.2 (37)	−3.9 ± 3.5 (37)	0.09
*P* value within group	0.001	<0.001	
Fasting plasma glucose (mg/dL)			
Baseline	133.5 ± 27.1 (37)	139.6 ± 20.2 (37)	0.28
Week 16	122.5 ± 19.5 (37)	125.2 ± 20.0 (37)	0.56
Change	−11.0 ± 20.2 (37)	−14.4 ± 16.9 (37)	0.44
*P* value within group	0.002	<0.001	
C-peptide (ng/mL)			
Baseline	2.1 ± 0.8 (37)	2.3 ± 1.1 (37)	0.34
Week 16	2.0 ± 0.8 (37)	2.1 ± 1.0 (37)	0.65
Change	−0.1 ± 0.5 (37)	−0.2 ± 0.5 (37)	0.29
*P* value within group	0.27	0.007	
Insulin (μIU/mL)			
Baseline	8.9 ± 5.5 (37)	10.3 ± 7.5 (37)	0.38
Week 16	7.3 ± 4.4 (37)	9.2 ± 7.4 (37)	0.20
Change	−1.6 ± 3.7 (37)	−1.1 ± 3.8 (37)	0.58
*P* value within group	0.012	0.08	
Glucagon (pg/mL)			
Baseline	164.6 ± 33.5 (37)	171.6 ± 42.4 (37)	0.43
Week 16	152.9 ± 24.5 (37)	159.3 ± 36.8 (37)	0.39
Change	−11.7 ± 31.2 (37)	−12.3 ± 31.6 (37)	0.93
*P* value within group	0.029	0.023	
HOMA-IR			
Baseline	3.1 ± 2.4 (37)	3.7 ± 3.2 (37)	0.34
Week 16	2.3 ± 1.5 (37)	3.0 ± 3.0 (37)	0.16
Change	−0.8 ± 1.9 (37)	−0.7 ± 1.5 (37)	0.73
*P* value within group	0.010	0.009	

Data are presented as mean ± standard deviation (*n*). *P* values show results for comparisons between groups by *t* test. *P* values within groups are results of paired *t* test

*HOMA-IR* homeostatic model assessment of insulin resistance. See Table 1 for other abbreviations

### Changes in clinical parameters of atherosclerosis and oxidative stress

Body weight and BMI decreased significantly in the dapagliflozin group compared with the metformin group (−1.9 ± 1.5 vs. −0.6 ± 1.3 kg; P < 0.001, −0.7 ± 0.6 vs. −0.2 ± 0.5 kg/m^2^; P < 0.001, respectively, Additional file 1: Table S1). With regard to lipid metabolism, total cholesterol and non-HDL cholesterol decreased significantly only in the metformin group, HDL cholesterol increased significantly only in the dapagliflozin group, LDL cholesterol increased significantly in both groups (Table 4). Triglyceride did not change in both groups. No significant changes were observed in serum adiponectin and apolipoprotein B48 in both groups. Compared to the metformin group, urine 8-OHdG/creatinine, a marker of oxidative stress, decreased significantly in the dapagliflozin group (Table 4).

**Table 4:** Changes in lipid parameters, markers of atherosclerosis, oxidative stress, and blood pressure

Parameters	Dapagliflozin group	Metformin group	*P* value
Total cholesterol (mg/dL)			
Baseline	201.8 ± 33.1 (35)	194.1 ± 31.9 (34)	0.33
Week 16	205.3 ± 36.0 (37)	182.2 ± 24.8 (37)	0.002
Percentage change (%)	2.2 ± 13.4 (35)	−5.4 ± 9.3 (34)	0.008
*P* value within group	0.35	0.002	
HDL cholesterol (mg/dL)			
Baseline	50.9 ± 9.0 (35)	53.4 ± 15.4 (34)	0.42
Week 16	55.1 ± 9.4 (37)	54.7 ± 15.2 (37)	0.90
Percentage change (%)	8.1 ± 13.4 (35)	3.4 ± 15.3 (34)	0.18
*P* value within group	0.001	0.20	
LDL cholesterol (mg/dL)			
Baseline	109.6 ± 33.2 (35)	96.1 ± 25.0 (34)	0.06
Week 16	124.0 ± 33.6 (37)	102.7 ± 26.0 (37)	0.003
Percentage change (%)	19.1 ± 28.0 (35)	9.7 ± 22.2 (34)	0.13
*P* value within group	<0.001	0.016	
Non-HDL cholesterol (mg/dL)			
Baseline	150.9 ± 32.8 (35)	140.7 ± 35.2 (34)	0.22
Week 16	150.2 ± 33.1 (37)	127.4 ± 24.7 (37)	0.001
Percent change (%)	0.9 ± 17.0 (35)	−7.7 ± 12.1 (34)	0.018
P value within group	0.74	<0.001	
Triglyceride (mg/dL)			
Baseline	142.8 ± 53.1 (37)	145.2 ± 69.0 (37)	0.87
Week 16	155.9 ± 81.1 (37)	136.2 ± 62.6 (37)	0.25
Percentage change (%)	11.3 ± 40.8 (37)	4.2 ± 53.4 (37)	0.53
*P* value within group	0.10	0.63	
Adiponectin (μg/mL)			
Baseline	2.6 ± 1.8 (37)	2.7 ± 1.8 (37)	0.77
Week 16	2.7 ± 2.2 (37)	2.8 ± 2.0 (37)	0.79
Change	0.1 ± 0.7 (37)	0.1 ± 0.7 (37)	0.97
*P* value within group	0.46	0.41	
ApoB48 (μg/mL)			
Baseline	4.8 ± 3.0 (37)	3.6 ± 1.9 (37)	0.036
Week 16	5.1 ± 3.9 (37)	3.1 ± 1.8 (37)	0.008
Change	0.3 ± 2.8 (37)	−0.4 ± 2.2 (37)	0.23
*P* value within group	0.55	0.24	
Urinary 8-OHdG/creatinine (ng/mg Cre)			
Baseline	4.6 ± 2.4 (36)	4.8 ± 2.0 (36)	0.62
Week 16	4.0 ± 1.9 (37)	5.8 ± 2.3 (37)	<0.001
Change	−0.6 ± 1.8 (36)	1.1 ± 2.2 (36)	<0.001
*P* value within group	0.047	0.004	
Systolic blood pressure (mmHg)			
Baseline	129.2 ± 13.7 (37)	130.0 ± 12.4 (37)	0.78
Week 16	126.4 ± 13.2 (37)	130.3 ± 13.5 (37)	0.21
Change	−2.8 ± 11.6 (37)	0.3 ± 12.6 (37)	0.27
*P* value within group	0.15	0.88	
Diastolic blood pressure (mmHg)			
Baseline	81.8 ± 9.6 (37)	79.9 ± 8.5 (37)	0.36
Week 16	80.0 ± 8.1 (37)	79.1 ± 7.5 (37)	0.61
Change	−1.8 ± 7.8 (37)	−0.8 ± 5.8 (37)	0.52
*P* value within group	0.16	0.41	

Data are mean ± standard deviation (*n*), or median [first quartile, third quartile] (*n*). *P* values show results for comparisons between groups by *t* test or Wilcoxon rank sum test. *P* values within groups are results of paired *t* test

*ApoB48* apolipoprotein B48, *8OHdG* 8-hydroxy-2′-deoxyguanosin. See Table 1 for other abbreviations

### Metabolic and hemodynamic changes

Significant increases in red blood cell count, hemoglobin, and hematocrit were noted in the dapagliflozin group (Table 5). In contrast to the above dynamic changes, white blood cell count remained unchanged, and platelet count decreased slightly in the dapagliflozin group and increased in the metformin group. Plasma uric acid decreased in the dapagliflozin group and was significantly different between the two groups (P < 0.001, Table 5). Analysis of data of patients with HbA1c ≥7.0% showed significant increases in red blood cell count, hemoglobin, and hematocrit and decrease in plasma uric acid in the dapagliflozin group (Table 6). Systolic and diastolic blood pressure were comparable between the two groups (Table 4).

**Table 5:** Changes in blood cell counts and uric acid

Parameters	Dapagliflozin group	Metformin group	*P* value
White blood cell count (/μL)			
Baseline	6378.6 ± 1553.5 (36)	6405.0 ± 1507.6 (36)	0.94
Week 16	6532.5 ± 1646.0 (35)	6372.6 ± 1700.5 (34)	0.69
Change	103.8 ± 1166.7 (34)	−106.2 ± 1183.0 (34)	0.46
*P* value within group	0.61	0.60	0.95
Red blood cell count (×10^4^/μL)			
Baseline	468.9 ± 40.2 (36)	468.3 ± 41.8 (36)	<0.001
Week 16	493.6 ± 41.6 (35)	456.6 ± 42.9 (34)	<0.001
Change	26.3 ± 16.3 (34)	−12.0 ± 17.5 (34)	
*P* value within group	<0.001	<0.001	
Hemoglobin (g/dL)			
Baseline	14.3 ± 1.3 (36)	14.4 ± 1.3 (36)	0.70
Week 16	14.9 ± 1.4 (35)	14.2 ± 1.7 (34)	0.05
Change	0.7 ± 0.5 (34)	−0.2 ± 0.9 (34)	<0.001
*P* value within group	<0.001	0.21	
Hematocrit (%)			
Baseline	42.6 ± 3.6 (36)	42.7 ± 3.3 (36)	0.94
Week 16	44.7 ± 3.5 (35)	41.8 ± 3.6 (34)	0.001
Change	2.2 ± 1.4 (34)	−0.9 ± 1.8 (34)	<0.001
*P* value within group	<0.001	0.006	
Platelet count (×10^4^/μL)			
Baseline	24.0 ± 6.4 (36)	24.0 ± 5.0 (36)	0.99
Week 16	23.9 ± 6.3 (35)	24.5 ± 4.7 (34)	0.64
Change	−0.3 ± 1.5 (34)	0.5 ± 2.2 (34)	0.09
*P* value within group	0.20	0.25	
Uric acid (mg/dL)			
Baseline	5.6 ± 1.1 (37)	5.8 ± 1.2 (37)	0.41
Week 16	4.9 ± 1.1 (36)	5.9 ± 1.1 (36)	<0.001
Change	−0.6 ± 0.7 (36)	0.1 ± 0.6 (36)	<0.001
*P* value within group	<0.001	0.43	

Data are mean ± standard deviation (*n*). *P* values show results of comparisons between groups by *t* test. *P* values within groups are results of paired *t* test

**Table 6:** Changes in various parameters in patients with HbA1c at baseline of ≥7.0%

Parameters	Dapagliflozin group	Metformin group	*P* value
Urinary 8-OHdG/creatinine (ng/mg Cre)			
Baseline	4.3 ± 1.7 (13)	4.4 ± 2.0 (17)	0.88
Week 16	4.1 ± 1.1 (13)	5.7 ± 2.4 (17)	0.021
Change	−0.2 ± 1.6 (13)	1.3 ± 2.3 (17)	0.042
*P* value within group	0.66	0.031	
Red blood cell (×10^4^/μL)			
Baseline	485.6 ± 32.2 (13)	463.5 ± 41.4 (16)	0.12
Week 16	507.9 ± 34.8 (12)	453.4 ± 30.9 (15)	<0.001
Change	21.3 ± 13.2 (12)	−14.7 ± 18.1 (15)	<0.001
*P* value within group	<0.001	0.007	
Hemoglobin (g/dL)			
Baseline	14.9 ± 0.8 (13)	14.0 ± 1.3 (16)	0.036
Week 16	15.6 ± 0.7 (12)	14.0 ± 1.9 (15)	0.008
Change	0.7 ± 0.4 (12)	−0.1 ± 1.3 (15)	0.030
*P* value within group	<0.001	0.67	
Hematocrit (%)			
Baseline	44.1 ± 2.3 (13)	41.7 ± 3.7 (16)	0.048
Week 16	46.1 ± 2.0 (12)	40.7 ± 3.0 (15)	<0.001
Change	2.0 ± 1.3 (12)	−1.4 ± 1.7 (15)	<0.001
*P* value within group	<0.001	0.007	
Uric acid (mg/dL)			
Baseline	5.9 ± 0.8 (13)	5.0 ± 0.9 (17)	0.012
Week 16	5.2 ± 0.6 (12)	5.3 ± 1.0 (17)	0.74
Change	−0.7 ± 0.7 (12)	0.3 ± 0.5 (17)	<0.001
*P* value within group	0.007	0.041	

Data are mean ± standard deviation (*n*). *P* values show results for comparisons between groups by *t* test or Wilcoxon rank sum test. *P* values within groups are results of paired *t* test or Wilcoxon signed rank test

### Adverse events

Table 7 lists the reported/observed AEs during the study. The recorded AEs were six for the dapagliflozin group and nine for the metformin group. There was no significant difference in the incidence of AEs between the two groups.

**Table 7:** Adverse events

	Dapagliflozin group (*n* = 40)	Metformin group (*n* = 40)	*P* value
Any adverse events	6 (15.0)	9 (22.5)	0.57
Hypoglycemia	1 (2.5)	0 (0.0)	1.00
Allergic rhinitis	0 (0.0)	1 (2.5)	1.00
Dry mouth	1 (2.5)	0 (0.0)	1.00
Esophageal varices hemorrhage	1 (2.5)^a^	0 (0.0)	1.00
Frequent urination	1 (2.5)	0 (0.0)	1.00
Upper respiratory tract infection	0 (0.0)	1 (2.5)	1.00
Interstitial lung disease	1 (2.5)^a^	0 (0.0)	1.00
Gastroesophageal reflux disease	1 (2.5)	0 (0.0)	1.00
Wound	1 (2.5)	0 (0.0)	1.00
Pharyngitis	0 (0.0)	1 (2.5)	1.00
Dyslipidemia	1 (2.5)	0 (0.0)	1.00
Decrease in appetite	0 (0.0)	1 (2.5)	1.00
Alopecia areata	0 (0.0)	1 (2.5)	1.00
Palpitation	0 (0.0)	1 (2.5)	1.00
Cervical dysplasia	0 (0.0)	1 (2.5)^a^	1.00
Lung cancer, stage 0	0 (0.0)	1 (2.5)^a^	1.00
Contusion	0 (0.0)	1 (2.5)	1.00
Diarrhea	0 (0.0)	1 (2.5)	1.00

Data are from treated set population

*P* values are results by Fisher exact test between groups

^a^Serious adverse event

## Discussion

Dapagliflozin is a relatively new oral glucose-lowering agent, and the DEFENCE study was designed to dissect the effects of dapagliflozin on endothelial function in T2DM patients with no history of cardiovascular diseases.

Several studies have already reported that the addition of dapagliflozin as monotherapy [24] or as an add-on to insulin [24, 25] was safe and effective in patients with inadequately controlled T2DM. Furthermore, not only the addition of dapagliflozin to metformin [26–28], but also initial combination therapy with both agents [29] resulted in improvement of glycemic control.

In this study, we planned to compare the effects of add-on dapagliflozin relative to those of metformin. Metformin is widely used worldwide and is known to provide protection against cardiovascular events [8]. A previous meta-analysis reported that there was no suggestion of increase risk for major adverse cardiovascular events with dapagliflozin compared with control [30]. However, to our knowledge, there is no published clinical trial that compared the effects of dapagliflozin add-on therapy to those of increased dose of metformin and little is known about the effects of the combination of dapagliflozin plus metformin on endothelial function. Thus, the DEFENCE study is the first study to address the effects of dapagliflozin on endothelial function as primary prevention of cardiovascular diseases.

T2DM is one of the major risk factors for progression of atherosclerosis and development of cardiovascular diseases [1, 2]. Recently, the EMPA-REG OUTCOME study reported that empagliflozin, another SGLT2 inhibitor, reduced cardiovascular mortality and hospitalization for heart failure among patients with T2DM [17]. It is noteworthy that empagliflozin reduced the number of patients who required admission to the hospital for heart failure, but not nonfatal ischemic cardiovascular events [17]. In the EMPA-REG OUTCOME study, most of the enrolled patients had history of cardiovascular diseases, and reduction in the primary outcome was observed at 3 months after the start of treatment with empagliflozin. Thus, it was concluded that the effect of empagliflozin on cardiovascular mortality is probably not related to the suppression of progression of atherosclerosis [17]. Another clinical trial; the DECLARE TIMI-58 study, which was designed to determine the effect of dapagliflozin on cardiovascular outcome, is ongoing but 40% of the enrolled patients had a previous cardiovascular event [31]. Thus, the efficacy of SGLT2 inhibitors as primary prevention of cardiovascular events and suppression of atherosclerosis remains unknown. In this regard, it is important to screen patients for the progression of atherosclerosis at an early stage in order to prevent cardiovascular events. Therefore, we set the inclusion criteria to include HbA1c ≥6.0 and <8.0% treated with only metformin 750 mg/day or one type of oral glucose-lowering agent in addition to 750 mg/day of metformin, and excluded patients with history of cardiovascular diseases. There is general agreement that vascular endothelial dysfunction is the initial stage of atherosclerosis, and considered the earliest predictor of future cardiovascular events in patients with T2DM [5]. Furthermore, FMD is recognized as a well-established surrogate marker of early endothelial dysfunction [32].

In the present study, there was no significant difference in ΔFMD between the two groups in FAS population. However, dapagliflozin add-on therapy significantly improved ΔFMD in patients with HbA1c ≥7.0% compared to metformin-increased therapy. In the dapagliflozin group, ΔFMD increased by 1.05 ± 2.59% after 16 weeks of treatment. In this regard, it is reported that an increase of 1% in FMD is associated with 12% reduction in adjusted relative risk of future cardiovascular events [33]. These results indicate that dapagliflozin add-on therapy provides better protection of endothelial function and prevention of future cardiovascular events in early-stage T2DM patients with inadequate glycemic control, compared to metformin-increased therapy. Because there was no significant difference in glycemic control between the two groups, it seems that the additional benefit of dapagliflozin is mediated through mechanisms other than its glucose lowering effect. To elucidate such mechanisms, we evaluated certain putative biomarkers that are considered to be associated with the progression of atherosclerosis. Interestingly, urine 8-OHdG, a marker of oxidative stress, was significantly reduced only in the dapagliflozin group. This result suggests that dapagliflozin reduces oxidative stress and this might contribute to the observed improvement in endothelial function. In fact, previous studies reported that elevated 8-OHdG was associated with progression of atherosclerosis [34] and diabetic vasculopathies [35]. In addition, experimental studies in rodents showed that SGLT2 inhibitors reduced urine 8-OHdG [36] and improved endothelial function [37]. These data support our hypothesis that dapagliflozin may improve endothelial function in T2DM by reducing oxidative stress. Interestingly, plasma uric acid was significantly reduced in the dapagliflozin group. Hyperuricemia can cause hypertension and vascular damage [38], and relates to cardiovascular diseases [39]. Thus, the observed decrease in serum uric acid could also play a role in the improvement of FMD in the dapagliflozin group. In addition, increases in red blood cell count, hemoglobin, and hematocrit were observed in the present study. SGLT2 inhibitors cause osmotic diuresis, and this contributes to changes in blood volume and blood cell kinetics [31]. Recently, increased hematocrit during SGLT2 inhibitors attracted attention as stimulus factor of erythropoiesis and oxygen transport to tissue as a protective role in cardiovascular diseases [40]. In this study, hematocrit was significantly increased in dapagliflozin group within not only FAS population analysis but also subgroup analysis of HbA1c <7.0 or ≥7.0%. Interestingly, a covariance analysis for ΔFMD by taking the change of hematocrit into account did not show any significant difference in subgroup of HbA1c <7.0 and ≥7.0% (P = 0.51 and 0.12, respectively). These data suggest that increased hematocrit might play a role on the increased FMD at least in part in dapagliflozin group. However, the Hisayama study showed both elevated and decreased hematocrit were associated with increased cardiovascular diseases [41]. Thus, it is still not clear whether the effect of dapagliflozin on hematocrit contributed to the improvement of FMD. Other markers of atherosclerosis, such as LDL cholesterol, adiponectin, and apolipoprotein B48 were comparable between the two groups.

### Limitations

Although these results clearly demonstrate the effectiveness of dapagliflozin add-on therapy, our study has several limitations. First, as this was an open-label design study, unexpected bias might occur because physicians knew their choice of treatment and subjects also knew the type of medications used. Second, the number of patients was relatively small and the duration of study was relatively short, so longer trials with larger sample size, preferably in subjects of different ethnicities, are needed. Third, subjects enrolled in this study had moderate hyperglycemia, so the effects of treatment in subjects with HbA1c of more than 8% remain unknown. Finally, we chose dapagliflozin 5 mg but not 10 mg. The results of phase III trial of dapagliflozin in Japanese patients with T2DM reported that both doses of dapagliflozin produced significant reduction in HbA1c from baseline. The adjusted mean change in HbA1c from baseline to 24 week was almost the same level (5 mg, −0.41% and 10 mg, −0.45%) [42]. In addition, another trial reported that dapagliflozin, initiated at 5 mg once daily and titrated, as needed, to 10 mg once daily, was well tolerated over 52 weeks in Japanese patients with T2DM [43]. The dose of dapagliflozin was 5 mg for initial treatment and it was up-titrated to 10 mg for the subjects whose HbA1c >7.5% after 12 weeks [43]. In the present study, according to inclusion criteria, basal HbA1c level was ≥6.0 and <8.0%, thus, the 5 mg of dapagliflozin was considered to be enough and well tolerated. Furthermore, 5 mg dapagliflozin costs a half price compared to 10 mg dapagliflozin. Thus, in general, the standard dose of dapagliflozin is considered 5 mg once daily in Japanese patients with T2DM.

## Conclusions

The DEFENCE study is the first to evaluate the effects of dapagliflozin on vascular endothelial function in T2DM. Dapagliflozin add-on therapy on metformin improved endothelial function assessed by FMD in patients with inadequate glycemic control. So far, there is no solid evidence that SGLT2 inhibitors play a protective role on endothelial function or can suppress the progression of atherosclerosis. The results of this study suggest that the combination therapy of dapagliflozin and metformin is a potential therapeutic option for the primary prevention of cardiovascular disease in patients with early-stage T2DM and moderate hyperglycemia.

## Additional file

**Additional file 1.** **Table S1.** Changes in body weight and BMI.

## Abbreviations

8-OHdG: 8-hydroxy-2′-deoxyguanosin
DPP-4: dipeptidyl peptidase 4
FMD: flow-mediated dilation
SGLT2: sodium glucose cotransporter 2
T2DM: type 2 diabetes mellitus
