# Supplementary material for: Effectiveness of dapagliflozin on vascular endothelial function and glycemic control in patients with early-stage type 2 diabetes mellitus: DEFENCE study
Source: Cardiovasc Diabetol. 2017 Jul 6;16:84. doi: 10.1186/s12933-017-0564-0 (PMC5500953; doi:10.1186/s12933-017-0564-0)
Supplement: Supplementary file 1 — Additional file 1. Table S1. Changes in body weight and BMI. [file 12933_2017_564_MOESM1_ESM.doc]

**SUPPORTING INFORMATION**

Additional Supporting Information for the online version of this article: Table S1

**List of 15 medical institutions participating in the study: (listed in alphabetical order):**

Haginaka Clinic

Haneda Bus-Road Clinic

Heiwa Hospital

Isuzu Hospital

Kawasaki Municipal Hospital

Kawasaki Saiwai Clinic

Keihin Hospital

Makita General Hospital

Oomorinaka Shinryojyo

Saiseikai Yokohamashi Tobu Hospital

Sasamoto Medical Clinic

Sato Hospital

Toho University School of Medicine

Tokyo Kamata Hospital

Wakaba Eye Hospital

| Table S1. Body weight and BMI | | | |
| --- | --- | --- | --- |
| Parameters | Dapagliflozin group | Metformin group | *P* value |
| Body weight (kg) | | | |
| Baseline | 73.9±14.3 (37) | 71.6±13.8 (37) | 0.48 |
| Week 16 | 72.0±14.8 (37) | 71.0±13.7 (37) | 0.76 |
| Change | -1.9±1.5 (37) | -0.6±1.3 (37) | <0.001 |
| *P* value within group | <0.001 | 0.005 |  |
| BMI (kg/m^2^) | | | |
| Baseline | 26.8±4.5 (37) | 26.3±3.5 (37) | 0.60 |
| Week 16 | 26.1±4.8 (37) | 26.0±3.6 (37) | 0.97 |
| Change | -0.7±0.6 (37) | -0.2±0.5 (37) | <0.001 |
| *P* value within group | <0.001 | 0.006 |  |
| Data are presented as mean ± standard deviation (*n*). *P* values show results for comparisons between groups by *t*-test. *P* values within groups are results of paired *t*-test. BMI, body mass index. | | | |
